# Associations between parental alcohol problems in childhood and adversities during childhood and later adulthood: a cross-sectional study of 28047 adults from the general population

**DOI:** 10.1186/s13011-021-00384-9

**Published:** 2021-06-07

**Authors:** Siri Håvås Haugland, Barbara Carvalho, Tonje Holte Stea, Arve Strandheim, John-Kåre Vederhus

**Affiliations:** 1 Department of Psychosocial Health, University of Agder, Postbox 422, 4604 Kristiansand, Norway; 2 Department of Health and Nursing Science, University of Agder, Kristiansand, Norway; 3 Department of Child and Adolescence Mental Health, Sørlandet Hospital, Kristiansand, Norway; 4 Regional Centre for Child and Youth Mental Health and Child Welfare, Norwegian University of Science and Technology, Trondheim, Norway; 5 Department of Child and Adolescent Psychiatry, Nord-Trøndelag Health Trust, Levanger, Norway; 6 Addiction Unit, Sørlandet Hospital, Kristiansand, Norway

**Keywords:** Family, Parents, Alcohol drinking, Adult survivors of child adverse events

## Abstract

**Background:**

Adverse childhood experiences (ACE) are related to adverse physical and mental health outcomes. However, few larger studies based on a general population sample with age groups ranging from young adults to elderly have investigated whether parental alcohol problems increase the risk of offspring subjective reports of ACE both during childhood and current adult adversities. The purpose of this study was to examine the associations between parental alcohol problems and adversities during childhood and later in adulthood.

**Methods:**

The 28,047 respondents were adults (> 18 years old) from the general population who participated in the Norwegian Counties Public Health Survey. The study had a cross-sectional design and included respondents’ evaluations of childhood experiences and current adult adversities. The short version of the Children of Alcoholics Screening Test (CAST-6, cut-off ≥3) measured parental alcohol problems. Multivariable logistic regression was adjusted for gender, age, and education.

**Results:**

Growing up with parental alcohol problems strongly increased the risk of experiencing a dysfunctional family environment during childhood (odds ratio [OR] 6.84; 95% confidence interval [CI] 6.36–7.36), perceiving childhood as difficult (OR 5.01; 95% CI 4.58–5.49), and reporting a lack of support from a trusted adult (OR 3.07; 95% CI 2.86–3.29). Parental alcohol problems were associated with a modestly increased risk of harmful alcohol use (OR 1.38; 95% CI 1.29–1.48), but the association with struggling with bad memories was strong (OR 4.56; 95% CI 4.17–4.98).

**Conclusions:**

Parental alcohol problems increased the risk of offspring experiencing adversities during both childhood and adulthood. Providing supportive services to these children and their families and addressing this issue as part of treatment is important to prevent alcohol related harm.

## Background

Excessive alcohol use has the potential to inflict collateral damage beyond the health problems experienced by the drinker, and there is a growing interest in studying alcohol’s harm to others [1]. Children living in families in which the parents drink excessively are particularly vulnerable to harmful consequences, as they possess little control over their own situation. For example, the children are more likely to experience adversities, such as a dysfunctional family environment, emotional abuse, violence, and neglect [2–7]. As one of the most prevalent adverse childhood experiences (ACEs), excessive parental alcohol use negatively affects a range of health outcomes [8] and may lead to long-term adverse outcomes, such as mental health problems and substance use as adults [2, 6, 7, 9, 10]. As ACEs may affect life differently because of the varying balance of risk factors and protective factors, it is relevant to map resilience factors, such as emotional support given by a safe and trusted grown-up [11, 12].

Screening for ACEs has traditionally included various types of experiences, mapping the specific ACEs considered to be most important and leaving out other adverse experiences. Previously published studies in this research area have also focussed on the quantity of experiences, and studies have indicated a cumulative effect of multiple ACEs with graded relationships to various health-related outcomes [13]. When trying to capture a person’s aggregated ACEs, parental alcohol abuse is typically included as part of a summed score reflecting ACE severity [14]. However, including parental alcohol abuse along with other ACEs cannot reveal the extent to which parental alcohol problems act as a risk factor for other ACEs [15]. Thus, investigating whether parental alcohol consumption may be associated with increased risk of other types of harmful life experiences is warranted.

Defining childhood as difficult is a strong predictor of adverse health outcomes in adulthood [16], and Graeber et al. [17] found that, regardless of the number of different types of adverse experiences reported, perceiving them as having a high impact and how the person rated this impact (positive or negative) were strongly associated with adult health-related quality of life. Approaching ACEs through self-reported items, in which respondents provide self-perceived descriptions of their childhood and the impact of difficult life experiences, could supplement previous research focussing on whether a respondent’s adverse experience was reported [17].

Studies on the short-term and long-term effects of living with parental alcohol misuse during childhood have often relied on college, university, or clinical samples, and these non-random samples may not be representative of the general population [11, 18]. A previous systematic review examining instruments measuring childhood adversities reported a lack of randomly selected samples representative of larger populations and a reliance on convenience samples [14]. Investigating parental alcohol problems and potential associations with other types of adversities in larger samples based on the general population could supplement existing findings.

The overall purpose of the present study was to investigate whether exposure to parental alcohol problems during childhood is associated with other adversities during childhood and adulthood. In addition, we wanted to address this issue based on data from a large sample drawn randomly from the general population. Specifically, we aimed to examine whether parental alcohol problems in childhood are associated with living in a dysfunctional family environment during childhood, perceiving childhood as difficult, a lack of accessible trusted adult during childhood, struggling with bad memories in adulthood, or harmful alcohol use in adulthood.

## Methods

### Participants and procedures

The present cross-sectional survey is part of the Norwegian Counties Public Health Survey (NCPHS)^1^ designed to provide information about health and health-related behaviours among adults at the community level across Norway. A random sample of 75,191 adult residents (≥18 years old) from all 30 municipalities in the southern region of Norway was drawn from the National Register (31.6% of the adult population in this region). After removing deceased persons, those registered in the Contact and Reservation Register with unverified contact information, or those with an address outside the included municipalities, a total of 61,611 residents were invited by SMS and e-mail to participate in this study during the autumn of 2019.

A total of 28,047 adults participated (response rate, 45.5%). Participants consented by filling out an online consent form and took approximately 15 min to complete the survey. Participants had the opportunity to withdraw from the study at any time.

### Measures

#### Sociodemographic variables

Education level was determined by the following question: “What is your highest completed level of education?” Response options were: 1) primary school (up to 10 years of education), 2) high school (up to 13 years of education), 3) university or university college (up to 16 years of education), 4) university or university college (16 years of education or more). The first option was defined as lower education, the second as middle level education, and options 3 and 4 were combined into higher education. Information about age and gender was retrieved from the national population register.

#### Cast-6

The short version of the Children of Alcoholics Screening Test (CAST-6) [19] was used to identify participants who had experienced parental alcohol problems during childhood. CAST-6 was developed from the original 30-item version [20]. This six-item measure was designed to assess whether participants perceive their parents’ alcohol consumption as problematic by asking the following questions: “Have you ever thought that one of your parents had a drinking problem?” “Did you ever encourage one of your parents to quit drinking?” “Did you ever argue or fight with a parent when he or she was drinking?” “Have you ever heard your parents fight when one of them was drunk?” “Did you ever feel like hiding or emptying a parents’ bottle of liquor?” “Did you ever wish that a parent would stop drinking?” Each item in the CAST-6 could be answered with Yes (1 point) or No (0 points). The total score for CAST-6 was the sum of the individual scores for each question; ≥3 was used as a cut-off for considering a respondent as having been exposed to parental alcohol problems. The validity of CAST-6 has been confirmed in adult populations, and the instrument has demonstrated high internal consistency (*r* = 0.92–0.94) and test-retest reliability (*r* = 0.93) compared to the original 30-item version (*r* = 0.93) using the recommended threshold of ≥3 [19, 21].

#### Assessment of other difficulties in childhood

This study includes four questions related to difficult childhood experiences originally developed for the HUNT Study (The Trøndelag Health study). The HUNT 3 wave [22] included a single item measuring difficult childhood, and this proved to be strongly related to negative health outcomes [16]. Participants were asked if they would describe their childhood as very good, good, average, difficult, or very difficult. For the analyses, the two latter response categories were combined into a “difficult childhood” category, and the others were treated as a reference group. The HUNT databank extended the number of questions about childhood in the fourth wave (HUNT4), and these were also included in the Agder Public Health Study. We assessed whether respondents perceived their childhood family environment to be dysfunctional by asking whether there were many arguments, conflicts, or turbulence, or whether communication was difficult in their childhood home. The response categories were: “to a very great extent”, “to a great extent”, “to a limited extent”, “to a very limited extent”, and “not at all”. The first two categories were combined and defined as “dysfunctional family environment” and the others were combined to a reference group.

We assessed whether there was an adult the respondents could seek support from and felt safe with during childhood. This question had the same response options as above, and those responding, “to a very limited extent”, “not at all” and “to a limited extent” were defined as having a “lack of an accessible trusted adult”. The others constituted a reference group.

#### Adverse outcomes in adulthood

We included a variable enabling participants to describe the extent to which they still struggled with bad memories from childhood due to loss, being let down, neglect, violence, ill treatment, or abuse. Response categories were the same as above. Those responding, “to a very great extent” and “to a great extent” were defined as “having bad memories”. The others constituted a reference group.

The World Health Organization (WHO) developed the 10-item Alcohol Use Disorders Identification Test (AUDIT) in the 1980s as a simple method of screening for excessive alcohol use [23]. We used the short 3-item version (AUDIT-C), which enhances feasibility when using the measure in large epidemiological surveys [24]. The AUDIT-C pertains to alcohol use patterns only and measures the frequency and quantity of alcohol use and the frequency of heavy episodic drinking. It performs as well as the full AUDIT in identifying heavy drinkers [24]. Early studies suggested a cut-off of ≥4 to achieve an acceptable ratio between sensitivity and specificity in order to avoid too many false positives [24]. Later studies recommended a slightly higher cut-off for men (≥5) but kept ≥4 for women [25, 26], and we applied this cut-off here.

### Statistical analysis

The sociodemographic characteristics of the sample are presented in Table 1. Chi-squared tests were used to identify differences in the prevalence of adverse outcomes between respondents who had been exposed to parental alcohol problems and those who had not (Table 2). Finally, multivariable logistic models were used to examine possible associations between parental alcohol problems and adverse outcomes. All models were adjusted for gender, age, and education level. Significance was defined as *p* < 0.05. All analyses were conducted in IBM SPSS version 25.

**Table 1:** Demographic data, Agder County Public Health Study (Norway 2019). *N* = 28,047

Demographic variables	n	%	Mean (SD), range
Female gender	14,925	53.2	
Age (years)			46.89 (16.30), 18–94
Education level			
Lower	3333	11.9	
Middle	11,088	39.7	
Higher	13,502	48.4

**Table 2:** Prevalence of adverse outcomes in relation to parental alcohol misuse status. Agder County Public Health Study (2019). *N* = 28,047

	Parental alcohol misuse (CAST-6 score > 3) (***n*** = 4346)	No parental alcohol misuse (CAST-6 score ≤ 3) (***n*** = 23,555)	***P***-value
**Difficult family environment**	2046 (47.1)	2677 (11.4)	<.001
**Perceived childhood as difficult**	1030 (23.7)	1308 (5.6)	<.001
**Lack of trusted adult during childhood**	1780 (41.1)	4284 (18.3)	<.001
**Struggle with bad memories**	1070 (24.7)	1494 (6.4)	<.001
**Harmful alcohol use**	1900 (43.9)	8342 (35.5)	<.001

*P*-values were obtained using the chi-squared test. Data are presented as n (%) unless otherwise noted

## Results

Slightly more women (53.2%) than men (46.8%) participated in this study. Almost half (48.4%) of the respondents reported a higher education level.

One in six respondents had a CAST-6 score > 3, indicating parental alcohol problems during childhood. A minority (8.4%) perceived their childhood as difficult or very difficult, but approximately one in six indicated that they grew up in a dysfunctional family environment. Lacking a trusted adult during childhood was more common, with more than one in five reporting inaccessibility to a trusted adult. Less than one in ten (9.2%) struggled with bad memories of violence, abuse, or neglect, and the most prevalent of our outcome variables was current harmful alcohol use among respondents (36.7%).

Table 2 shows an unadjusted comparison between those reporting parental alcohol problems versus those without this experience. Those who were exposed to parental alcohol problems were > 4-times more likely to perceive their childhood as difficult (23.7% versus 5.6%). Growing up in a dysfunctional family environment was more than 4-times more common among respondents exposed to parental alcohol problems, and more than twice as many respondents among those who grew up with parental alcohol problems reported lack of access to a trusted adult during childhood compared to the other respondents (41.1% versus 18.3%). Those who grew up with parental alcohol problems were 4-times more likely to struggle with bad memories of abuse/neglect/violence (24.7% vs. 6.4%). We found only a modest 8.4% higher proportion of respondents with harmful alcohol use among those who had experienced problematic parental alcohol use compared to those without the exposure (43.9% versus 35.5%, Table 2).

**Fig. 1:**
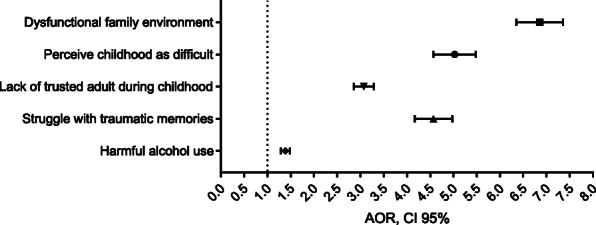
Multivariable logistic regression. Visualization of the adjusted (for gender, age, and education) estimates of associations between parental alcohol problems and adversities during childhood and adulthood based on the Agder County Public Health Study (Norway 2019). Data are presented as adjusted odds ratios and 95% confidence intervals

The results from multivariable analyses of associations between parental alcohol misuse during childhood and the various adverse outcomes adjusted for gender, age, and education are given in Table 3. Effects estimates (odds ratio [OR] and 95% confidence interval [CI]) for the associations between exposure and outcomes are visualized in a forest plot in Fig. 1.

**Table 3:** Multivariable logistic regression^a^ of associations between parental alcohol problems and adversities during childhood and adulthood. Agder County Public Health Study (2019). *N* = 28,047

	Dysfunctional family environment	Perceive childhood as difficult	Lack of trusted adult during childhood	Struggle with bad memories	Harmful alcohol use
**Parental alcohol problems**	6.84 (6.36–7.36)***	5.01 (4.58–5.49)***	3.07 (2.86–3.29)***	4.56 (4.17–4.98)***	1.38 (1.29–1.48)***
Gender (ref: male)	1.50 (1.40–1.61)***	1.50 (1.37–1.64)***	1.01 (0.95–1.07)	1.65 (1.50–1.80)***	0.95 (0.90–1.00)*
Age	0.98 (0.98–0.98)***	0.98 (0.98–0.98)***	1.00 (1.00–1.00)*	0.98 (0.98–0.98)***	0.97 (0.97–0.97)***
Lower education	1.36 (1.23–1.51)***	2.23 (1.97–2.53)***	1.75 (1.60–1.91)***	2.10 (1.86–2.39)***	1.10 (1.02–1.20)*
Middle education	0.97 (0.90–1.04)	1.30 (1.18–1.44)***	1.25 (1.18–1.33)***	1.31 (1.19–1.43)***	1.18 (1.12–1.24)***
Higher education	ref	ref	ref	ref	ref

^a^Adjusted for gender, age, and education level

Data are presented as odds ratios with 95% confidence intervals. * *p* < 0.05, *** *p* < .001

Growing up with parental alcohol problems was clearly associated with a retrospective perception of a childhood characterized by challenging cirumstances, such as a dysfunctional family environment (OR 6.84; 95% CI 6.36–7.36), describing childhood as difficult (OR 5.01; 95% CI 4.58–5.49) and lacking access to a trusted adult as a child (OR 3.07; 95% CI 2.86–3.29),). We also found a strong association between experiencing parental alcohol problems in childhood and struggling with bad memories, such as of abuse, neglect, or violence, as an adult (OR 4.56; 95% CI 4.17–4.98). Growing up with parental alcohol problems only modestly increased the odds of respondents reporting their own harmful alcohol consumption (OR 1.38; 95% CI 1.29–1.48). As this association was weaker than expected, we performed sensitivity analyses with a higher cut-off (> 8 for both genders), as well as with a lower cut-off (3 for women and 4 for men), without markedly changing the result (results not shown).

## Discussion

In this study, we investigated whether growing up with parental alcohol problems is associated with other adversities during childhood and later in adulthood. We revealed a very strong association between parental alcohol problems and experiencing a dysfunctional family environment characterized by arguments, tension, conflicts, or poor communication. These findings confirm previously published studies showing that parental alcohol problems can have a negative impact on family dynamics [27–29]. When circumstances around a child are difficult, the presence of other protective factors may buffer the impact of adverse experiences [11, 12]. However, our findings suggest that adults who report to have parents who drank excessively during childhood also were more likely to lack access to a trusted adult from whom they could seek support when they were young. Adults with experiences with parental alcohol problems more often described their childhood overall as difficult, which is in line with findings reported by other studies [5, 7].

Excessive alcohol use may impair parents’ ability to create a safe environment for their children and increase the risk of emotional and physical abuse and child neglect [4, 5, 7, 30, 31]. Such experiences may remain a burden as children become adults. Our study corroborated these findings; adults who had experienced parental alcohol problems in childhood had strongly increased odds of struggling with bad memories due to loss, being let down, neglect, violence, ill treatment, or abuse. This concurs with the study by Hall and Webster, who found that young adult children of parents with alcohol problems had enhanced traumatic event symptomatology compared to adults who, as children, experienced traumatic events other than parental alcohol problems and those who indicated neither problem [32].

Surprisingly, the association between parental alcohol problems in childhood and harmful alcohol use in adulthood were modest and the weakest of all associations in our study (OR = 1.38). This contrasts the study by Anda et al. [5], who found a strong relationship between parental alcoholism and personal alcoholism (OR = 3.5/4.5 for father/mother alcoholism and OR = 5.6 for both parents). Notably, our study did not apply personal alcoholism as an outcome, but a frequency-quantity measure (AUDIT-C). The high percentage of harmful alcohol use in our study indicates that our group of respondents did not constitute merely persons with an alcohol use disorder. Previous Norwegian studies have found a significantly lower lifetime prevalence of alcohol dependence or abuse (9.4 and 22.7% [33]) than the proportion of respondents in our study with harmful alcohol use. However, when we re-examined our analysis with a stricter cut-off (AUDIT-C ≥ 8), which would capture a group with more heavy drinking, the strength of the estimate remained modest, indicating a contrary finding to Anda et al.’s study [34]. The difference may be explained by the sample differences; Anda et al. examined a clinical sample of adults who visited a primary care clinic, whereas we examined a general population sample.

Whether problematic parental alcohol use has a causal inference for adult alcohol use in offspring cannot be determined by the present study. However, a systematic review suggested that parental drinking predicts drinking behaviour in offspring during adolescence [35]. Anda et al. [5] showed that the risk of developing an alcohol use disorder increases with the number of adverse experiences in childhood, and our data support the first part of his findings. A Swedish adoption study [36] shows that substance use disorder is an etiologically complex phenomenon that is influenced by both genetic risk factors and environmental factors, and the interactions between these. The increased risk of harmful alcohol use found among our respondents with parents with alcohol problems may therefore partly be explained by genetic disposition.

### Methodological considerations

Although we included retrospective information about childhood, our study was, in principle, cross-sectional, and the usual caveat about a cross-sectional design must be considered. Findings should be interpreted with caution with regards to causality. Research within the ACE-field has shown that childhood adversities are often interrelated [15], and we cannot out rule the possibility of the co-existing factors that may contribute to explain the association between parental alcohol use and the adverse outcomes in our study.

The large sample drawn randomly from a general population including all age groups > 18 years provided an opportunity to examine the relationship between parental alcohol problems and adverse outcomes, adding to existing knowledge based on smaller clinical studies [14]. The measures used to assess adverse life experiences included items about self-perceived severity, which added to the previous methodology focussing on specific types and quantity of experiences [14].

Although a previous review showed that retrospective reports of ACEs among adults include a substantial number of false negatives and substantial measurement error, false positive reports were probably rare [37]. Therefore, our study may be hampered by under-reporting, which may have led to an underestimation of the effects. Although some studies have found some bias in retrospective reports, Hardt and Rutter [37] claimed that the bias is not sufficient to invalidate retrospective reports in general. Whether parents recovered from their alcohol problems was not possible to take account for in this study.

## Conclusion

Our study shows that exposure to parental alcohol problems during childhood is strongly associated with both difficult life experiences during childhood and adverse outcomes later in adulthood. As parental alcohol problems appear to be quite prevalent and, therefore, affect many families, providing support and treatment opportunities to individuals with alcohol problems is central to reducing the alcohol-related harm to both the drinker and the offspring. Additionally, practitioners should provide specific support to children and work with the social network to ensure that children have trusted adults available.

## Data Availability

The data were provided by the NIPH by permission. NIPH will make data available in a repository (https://helsedata.no/en/) upon application.

## Abbreviations

OR: Odds ratio
CI: Confidence Interval
CAST-6: Children of Alcoholics Screening Test (short version with 6 items)
NCPHS: Norwegian Counties Public Health Survey
ACE: Adverse childhood environment
HUNT: The Nord-Trøndelag Health Study
AUDIT: Alcohol Use Disorders Identification
SPSS: Statistical Package for the Social Sciences
REK: The National Committees for Research Ethics in Norway
NIPH: Norwegian Institute of Public Health

## Footnotes

1 https://www.fhi.no/div/helseundersokelser/fylkeshelseundersokelser/
